# Prorenin in Hepatic Stellate Cell Extracellular Vesicles Induces Platelet‐Dependent Thrombin Formation and Release of Profibrotic TGF‐β

**DOI:** 10.1096/fj.202501902R

**Published:** 2025-10-15

**Authors:** Rui Chen, Emily Huang, Xianfang Wu, Megan R. McMullen, Xianjia Wang, Laura E. Nagy, Ge Jin, Scott J. Cameron, Thomas M. McIntyre

**Affiliations:** ^1^ Department of Cardiovascular & Metabolic Sciences Cleveland Clinic Research, Cleveland Clinic Cleveland Ohio USA; ^2^ Department of Inflammation and Immunity Cleveland Clinic Research, Cleveland Clinic Cleveland Ohio USA; ^3^ Department of Infection Biology Cleveland Clinic Research, Cleveland Clinic Cleveland Ohio USA; ^4^ Department of Molecular Medicine Case Western Reserve University School of Medicine Cleveland Ohio USA; ^5^ Rammelkamp Center for Research, and Department of Medicine Case Western Reserve University School of Medicine and MetroHealth System Cleveland Ohio USA; ^6^ Department of Cardiovascular Medicine Cleveland Clinic Cleveland Ohio USA

## Abstract

Dysregulated coagulation and platelet activation contribute to liver dysfunction and fibrosis, but mechanisms initiating these events are undefined. The hepatic stellate cell (HSC) agonist Concanavalin A (ConA) rapidly induces hepatitis, which progresses to hepatic fibrosis after serial exposure for 8 weeks. Extravascular platelets were intercalated throughout normal liver parenchyma, with ConA treatment activating resident platelets to degranulate and display P‐selectin. HSCs matured from activated human induced pluripotent stem cells or the human LX‐2 cell line released extracellular vesicles (EVs) that stimulated platelet aggregation, yet these particles lacked known platelet agonists, and this response differed from established behaviors. Enzymatic assays, biologic, chemical and aptamer inhibitors, immunohistochemistry, qPCR, REN siRNA, and western blotting elucidate a novel HSC‐EV mediated pathway to platelet activation. The aspartyl protease inhibitor pepstatin, or specific inhibitors Aliskiren and VTP23999 of the aspartyl protease renin suppressed HSC EV‐induced platelet activation, as did siRNA knockdown of prorenin. HSC maturation from mesenchymal cells increased prorenin transcripts, and HSC‐EV contained surface‐associated prorenin. Platelets expressed the prorenin receptor that overcomes prorenin auto‐inhibition, and HSC EV interacting with platelets generated renin peptidolytic activity. This interaction stimulated extrinsic and common coagulation cascades, formation of thrombin over time, activated platelet PAR‐1 thrombin receptors, and induced robust TGF‐β release. This TGF‐β stimulated the LX‐2 cell TGFbR1 receptor, Smad phosphorylation, and profibrotic protein expression. We identify hitherto undiscovered pathways by which platelet and HSC interaction stimulates thrombosis through prorenin activation and show intrahepatic platelets are positioned to stimulate fibrotic protein deposition in a model of hepatic fibrosis.

## Graphical abstract

Platelets interact with extracellular vesicles shed by hepatic stellate cells to activate both cell types. Extracellular vesicles shed by activated hepatic stellate cells contain inactive prorenin. HSC‐EV interact with quiescent platelets to non‐proteolytically activate ligated prorenin. This enables time‐dependent organization of coagulation protease complexes on platelets that generate active thrombin. Thrombin activates platelet PAR‐1, induces robust platelet activation and aggregation with TGF‐β release. Platelet‐derived TGF‐β stimulates HSC fibrotic protein expression and deposition.

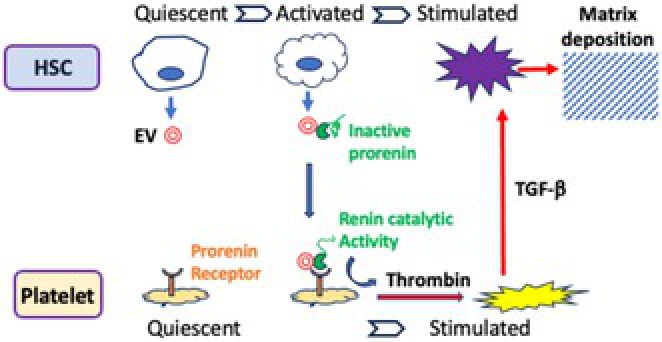

**Abbreviations**
BMP4: bone morphogenic protein‐4
CD62: cluster designation 62; P‐selectin
ConA: Concanavalin A
DAB: 3, 3′‐diaminobenzidine
DRI: direct renin inhibitor
EndoF: endoglycosidase type F; peptide‐*N*‐glycosidase F
EV: extracellular vesicle
FBS: fetal bovine serum
FXa: factor X‐activated
HBSS: Hepes‐buffered salt solution
HRP: horseradish peroxidase
HSC: hepatic stellate cell
iPSC: induced pluripotent stem cells
MEM: minimum essential medium
PAR‐1: protease‐activated Receptor‐1
PGE_1_
: prostaglandin E_1_
RAS: renin‐angiotensin system
RPMI: Roswell Park Memorial Institute Medium
RT‐PCR: reverse transcription polymerase chain reaction
SDS‐PAGE: sodium dodecyl sulfate‐polyacrylamide gel electrophoresis
Smad: suppressor of mothers against decapentaplegic
TF: tissue factor
TFPI: tissue factor pathway inhibitor
TGFbR1: tumor growth factor beta receptor 1
TGF‐β: tumor growth factor‐beta

## 1 Introduction

Liver fibrosis and cirrhosis create enormous healthcare needs [1], with increased mortality risk for patients unable to undergo orthotopic liver transplant. Hepatic insults including toxins, steatosis, and inflammation stimulate hepatic stellate cell (HSC) synthesis and deposition of fibrillar extracellular matrix, which fate tracing shows to be the primary fibrotic cell in models of liver disease [2]. Coagulation and thrombosis are key contributors to hepatic fibrosis [3] and platelets, a prolific source of a myriad of stimulatory and mitogenic factors [4], accumulate within liver parenchyma during hepatic inflammation [5, 6] to support TGF‐β‐dependent development of liver disease [7, 8]. Platelets contain ~100 times more TGF‐β than non‐neoplastic tissues [9] and are the primary source of soluble blood‐borne TGF‐β [10]. Platelet TGF‐β stimulates the type I TGF‐β receptor (TGFRb1), and the downstream Smad transcription factors it activates [11, 12], to induce expression and release of pro‐fibrotic extracellular matrix proteins by HSC. Accordingly, platelet inhibition or depletion reduces injury‐induced hepatitis and fibrosis in mice [11, 13, 14], while anti‐platelet therapy significantly reduces the odds of developing hepatic fibrosis in man [15].

Dysregulation of the coagulation cascade is a hallmark of a failing liver that contributes to hepatic fibrosis [16, 17]. Thrombin generation by either the Tissue Factor‐dependent extrinsic or the contact‐initiated intrinsic coagulation cascades is tightly regulated [18] to prevent liver injury. The final steps in thrombin formation by the common coagulation pathway downstream of these two initiating cascades are catalyzed by “tenase” and prothrombinase multi‐component complexes organized on the surface of activated platelets [19]. Thrombin is the single complete platelet agonist [20], stimulating human platelets through protease‐activated receptor‐1 (PAR1) signaling [21, 22]. This receptor is also expressed by HSCs [23], and inhibition of PAR1 protects against experimental liver fibrosis [23, 24]. While activated thrombin and activated platelets clearly contribute to hepatic injury, the way either inactive thrombin zymogen or platelets are initially activated to promote fibrosis is undefined.

Renin, a pepsin‐like aspartyl protease, and its enzymatically inactive prorenin zymogen are expressed by non‐renal cells [25] independent of the primary role of renin in regulating blood pressure [26]. The way renin expression is regulated is unknown [27], but HSC from normal individuals do not express detectable amounts of renin yet do so when isolated from cirrhotic human liver [28, 29]. The active sites and their catalytic mechanism of pepsin‐like aspartic proteases are unique [30], with the first‐in‐class, FDA‐approved Direct Renin Inhibitor (DRI) Aliskiren designed to specifically intercalate into the renin active site [31] to control blood pressure. Unexpectedly, Aliskiren also suppresses experimental liver injury [32, 33, 34, 35], as well as experimental venous [36] and arterial [37] thrombosis, suggesting an unidentified role(s) for renin in hepatic dysfunction and dysregulated thrombosis.

Western blotting and ELISA show circulating plasma prorenin exceeds renin concentration by three to four orders of magnitude and that renin abundance is modulated independently of prorenin [38]. Since circulating prorenin is not converted to renin and does not alter blood pressure [39], these proteins behave as distinct entities. However, prorenin zymogen, and to a lesser extent its renin product, are both ligands for the signaling prorenin receptor (p)RR [40], with inhibition or knockdown of (p)RR attenuating diet‐induced hepatic fibrosis [41]. The pro domain of prorenin intercalates into renin's active site, blocking substrate access [42, 43], but (p)RR ligation of inactive prorenin induces a conformational alteration that displaces the pro domain from the active site to relieve this self‐inhibition [43]. Renin enzymatic activity from this non‐proteolytic activation is then susceptible to Aliskiren inhibition [44, 45].

We identify resident platelets in murine liver parenchyma that have been activated by serial exposure to the HSC agonist Concanavalin A (ConA) that induced hepatic fibrosis; we identify a novel route to platelet activation through prorenin presented by HSC‐derived extracellular vesicles (EV), and we show this intercellular interaction initiates the extrinsic coagulation cascade, thrombin formation, PAR1 signaling, and TGF‐β release from activated platelets that stimulates fibrotic protein expression by HSC.

## 2 Materials and Methods

### 2.1 Experimental Design

We assessed platelet activation by incubating freshly isolated washed human platelets with media conditioned by hepatic stellate cells, EV isolated from these cells, or established soluble platelet agonists by aggregometry. We assayed platelet responses over time using blood obtained from multiple separate donations to obviate the wide variation in platelet reactivity among individuals. The figures presented generally are from a single experiment with platelets from a single donor, with the stated number of experiments from other blood donors presented as biologic replicates. However, in some panels where a tested reagent was without effect in three experiments, a single representative trace is included as a composite representation.

### 2.2 Ethics Statement

Human experiments were conducted according to the 2003 Declaration of Helsinki principles, with all human samples obtained in accordance with an approved protocol by the Cleveland Clinic Institutional Review Board. Each blood donor received information sufficient to determine whether to donate blood and provided written informed consent prior to the blood draw. Inclusion and exclusion criteria for this study were any healthy adult males or females not taking any medications who have not imbibed caffeinated beverages nor taken aspirin in the four days prior to the blood draw. Patient demographics, including age, sex, or weight, were not a component of the study and were not assessed. Consent is obtained for a single blood draw, and so there is no attrition from this study, nor were statistical analyses of the patient population relevant as patient demographics were not assessed as a group. Study participants' names and other Health Insurance Portability and Accountability Act (HIPAA) identifiers were removed from all data. The protocols for the use of mice were approved by the Cleveland Clinic IACUC.

### 2.3 Chemicals and Reagents

Endotoxin‐free human serum albumin (25%) was from Baxter Healthcare. Thrombin and collagen for aggregation were from Chrono‐log (Havertown, PA), while media and sterile filtered HBSS were prepared by the Cleveland Clinic Research Institute media preparation core. LX‐2 cells (RRID:CVCL_5792) were from Sigma‐Aldrich (St. Louis, MO), HEPG2 and HEP3b were from ATCC (Gaithersburg, MD), and HEK blue‐TGFβ (hkb‐tgfbv2) were from InvivoGen (San Diego, CA). Cell lines were reestablished from an early passage after 20 passages, with contamination assessed halfway through this time by STR (ATCC) analysis. Size filters (3 kDa) were from EMD Millipore (Billerica, MA). Gradient (4%–20%) SDS‐PAGE gels were from BioRad (Hercules, CA); Halt protease inhibitor mix (containing Pepstatin A and EDTA) was from Thermo Scientific (Rockford, IL); PVDF membranes were from MilliporeSigma (St. Louis, MO), and ECL reagent was from ThermoFisher. TFPI and A83‐01 were from R&D Systems (Minneapolis, MN); GGACK and PKSI‐527 from SantaCruz Biotechnology (Dallas, TX); Dabigatran, PPACK, Aliskiren, VTP27999, and EW‐7197 from Cayman Chemical (Ann Arbor, MI); Vorapaxar and Edoxaban from Selleck Chemicals (Houston, TX). Cell Signaling Technology (Burlington, MA) provided TGF‐β, and Vector Laboratory (Burlingame, CA) provided mounting media. The thrombin aptamer, its scrambled control, and Factor 9 aptamer were synthesized by IDT (Coralville, IA), while human renin 27mer siRNA (SR304432) construct B and the universal scrambled negative control duplexes were from Origene (Rockville, MD). Antibodies against pAKT273, AKT, collagen 1 Coralite594, TGF‐β, pSMAD2, renin/prorenin, and Tissue Factor were from Cell Signaling Technologies (Boston, MA); β‐actin from Santa Cruz Biotechnology (Dallas, TX); laminin, collagen 1‐Alexa488, fibronectin Alexa488, prothrombin, and (p)RR from Abcam (Cambridge, United Kingdom). The thrombin inhibitor Phe‐Pro‐Arg‐chloromethyl ketone (FPR‐CMK) was from Hemalogic Technologies (Essex Junction, MA). Thrombin, PGE_1_, sodium dodecyl sulfate (SDS), chymostatin (a mixture of A, B, and C peptides), kallikrein, PAR1 peptide TRAP6, thrombin benzoyl‐FVR‐AM, N‐glycanase F, renin assay kit, and all other reagents were from Sigma.

### 2.4 Murine Concanavalin A Fibrotic Model

Male C57BL/6J mice (RRID:IMSR_000664) [females were not used as they vary markedly in their responses to ConA [46]] were purchased from Jackson Laboratories and housed in shoebox‐sized cages (four animals per cage) with microisolator lids in the Cleveland Clinic Biological Resources Unit. Mice were age‐ and weight‐matched and then randomized into saline and ConA groups and then injected once weekly through their tail vein with saline (*n* = 4) or 12 mg/kg ConA (*n* = 11, with 3 deaths) for eight weeks [47]. Mice were euthanized 24 h after the last injection.

### 2.5 Immunohistochemistry

Formalin‐fixed paraffin‐embedded liver sections were cut into 5 μm sections for staining. Slides were de‐paraffinized and stained with Pico Sirius Red (Direct Red 80, 36‐554‐8, Sigma Aldrich, St. Louis, MO) (RRID:SCR_008988) for the detection of collagen fibers. Detection of TGFβ1 (1:200, ab215715, Abcam, Cambridge, UK) and P‐selectin (1:2000, ab316113, Abcam) after de‐paraffinization; heat antigen retrieval was performed with Tris‐EDTA pH 9.0 or Citrate buffer pH 6.0 for CD61 (1:400, MA5‐32077, Invitrogen, Carlsbad, CA). Endogenous peroxidase was blocked with 3% hydrogen peroxide for 20 min before blockade with normal goat serum prior to overnight incubation with primary antibodies. Vectastain elite ABC HRP kit (PK6101, Vector Laboratories, Newark, CA) and DAB substrate (SK‐4100, Vector Laboratories) were used according to the manufacturer protocol to visualize staining. Hematoxylin was used for nuclear staining. Liver sections were coded at the time of collection to assure an unbiased analysis, and at least 3 images were acquired per section at 10× or 20× on the Keyence BZX‐700 microscope (Keyence, Itasca, IL). Semi‐quantification of positive staining was performed using Image J (RRID:SCR_003070) (NIH, Bethesda, MD). Results were displayed as percent positive staining of total tissue area. Unpaired *t*‐tests were used to test for statistical significance between treatment groups.

### 2.6 Ex Vivo Matrix Protein Immunohistochemistry

Human LX‐2 HSC were grown in 8 well glass chamber slides and incubated for 24 h in the presence or absence of either A83‐01 or EW 7197 TGFbR1 inhibitors with media conditioned by prior co‐incubation of platelets with HSC‐EV. These wells were rinsed thrice with PBS prior to the addition of 150 μL Cytofix/Cytoperm fixation and permeabilization solution (BD Biosciences) for 30 min at 4°. These wells were then washed thrice with BD Biosciences Perm/Wash buffer before being blocked with 10% goat serum in Perm/Wash buffer for 60 min at 4°. This was removed and the wells were then incubated overnight with 100 μL of Perm/Wash buffer containing primary antibody, 1:200 CoraLite 594‐conjugated collagen Type I antibody, Proteintech CL594‐67288, or 1:100 Alexa Fluor 488‐conjugated Abcam fibronectin. These wells were washed thrice with PBS and mounted with DAPI mounting media.

### 2.7 Platelet Purification and Analysis

Human blood was drawn into acid‐citrate‐dextrose and centrifuged (200 × *g*, 20 min) to obtain platelet‐rich plasma. Purified platelets were prepared from this platelet‐rich plasma as in the past, where platelet‐rich plasma was filtered through two layers of BioDesign 5‐μm CellMicroSieves mesh (Thermo Fisher Scientific, Waltham MA) to remove nucleated cells before re‐centrifugation (520 × *g*, 30 min) in the presence of 100 nM PGE_1_. Braking was not used in any centrifugation procedure. The resulting pellet was resuspended in 50 mL PIPES/saline/glucose (5 mM PIPES, 145 mM NaCl, 4 mM KCl, 50 μM Na_2_HPO_4_, 1 mM MgCl_2_, and 5.5 mM glucose) containing 100 nM PGE_1_, before these cells were centrifuged (520 × *g*, 30 min) to remove residual plasma protein and PGE_1_. The recovered platelets were resuspended in 0.5% human serum albumin in Hank's Balanced Salt Solution (HBSS). Washed platelets (2.5 × 10^8^/mL) were stimulated with 0.02–0.05 U thrombin (with the minimally active amount determined daily for each donor), in the presence of 1%–2% autologous platelet poor plasma, as with tumor‐derived microvesicles [48], by recording changes in transmittance (Chrono‐Log 700) in 100 μL cuvettes stirred at 1000 rpm. Here, complete transmittance was defined by the absorbance of platelet poor plasma. Platelet response to HSC‐derived EV results in hysteresis prior to the initiation of aggregation and aggregation; but once aggregation is initiated, this process is always complete. Since the abundance of EV determines the length of this delay, inhibition is measured as a change in the time for 50% activation of the platelet population, with 0% inhibition at the time of 50% aggregation and 100% at the end of the 60 min incubation period unless stated otherwise.

### 2.8 Differentiation of hPSC‐Derived Quiescent Hepatic Stellate Cells

Human induced pluripotent stem cells (hiPSCs, hiPSC‐W3 line) were cultured on growth factor‐reduced Matrigel according to the manufacturer's instructions, using a feeder‐independent, mTeSR1‐based medium (Stemcell Technologies, Vancouver, Canada). Cultures were replenished with fresh medium daily, and cells were passaged every 4–6 days as clumps using ReLeSR (Stemcell Technologies). For the experiments described in this study, hiPSCs between passages 30 and 40 were used. For differentiation toward quiescent qHSCs, culture plates were coated twice with matrigel to enhance the thickness of the protein layer. hiPSC‐W3 cells were first dissociated into a single‐cell suspension and seeded onto matrigel‐coated plates in mTeSR1 medium supplemented with ROCK inhibitor (Y‐27632, final concentration 10 μM), targeting approximately 20% confluence by the following day. Mesoendoderm differentiation was initiated by treating hiPSCs with RPMI/B‐27 medium (RPMI 1640 supplemented with 2% B‐27 minus insulin, 0.5% GlutaMAX, and 0.5% non‐essential amino acids) containing 10 μM CHIR99021 for one day. Cells were then treated with RPMI/B‐27 supplemented with 20 ng/mL BMP4 for an additional three days to promote mesoderm induction. Alternatively, mesodermal cells were derived using Mesoderm Induction Medium (Stemcell Technologies) following the manufacturer's protocol. To induce further differentiation of mesodermal progenitors, cells were cultured in RPMI/B‐27 supplemented with 50 μg/mL ascorbic acid, 0.5% ITS, 0.5 μM dexamethasone, 5 ng/mL BMP4, and 20 ng/mL FGF1 for three days. Finally, quiescent HSCs were generated by culturing cells in RPMI/B‐27 supplemented with 50 μg/mL ascorbic acid, 0.5% ITS, 0.5 μM dexamethasone, 1% synthetic lipids, 30 ng/mL EGF, 10 ng/mL FGF2, and 5 μM retinol for six days. A schematic diagram of the differentiation protocol is presented in Figure 2B. To induce HSC activation, the qHSCs generated above were detached, dissociated into a single‐cell suspension, and seeded onto culture plates in DMEM supplemented with 10% fetal bovine serum (FBS). Cells were maintained for 7 days, with passaging performed upon reaching 100% confluency.

**FIGURE 1:**
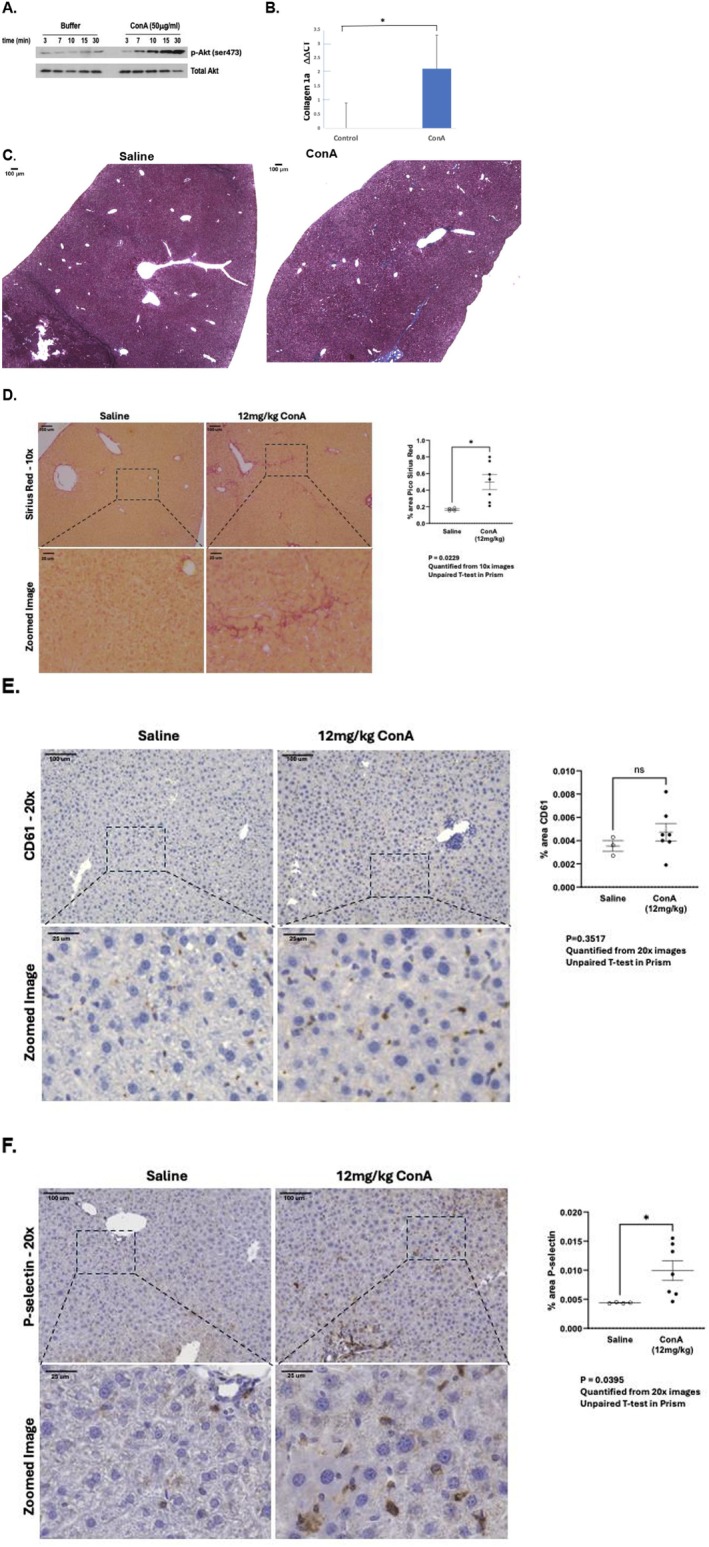
ConA is an HSC agonist with serial exposure inducing hepatic fibrosis and intrahepatic platelet activation. (A) ConA is a rapid LX2 cell agonist. LX2 cells were treated with ConA (50 μg/mL) and at the specified times aliquots were solubilized and then western blotted with anti‐phospho AKT or anti‐AKT with detection by HRP‐conjugated secondary antibody. *n* = 3. (B) Hepatic collagen 1a mRNA increases in mice serially exposed to ConA. ΔΔCt of collagen 1a was quantified in homogenized unprocessed tissue samples by RT‐PCR from mice injected weekly with saline or 12 mg/kg ConA. The *p* value of the triplicate samples (*p* = 0.0119) was less than 0.05 denoted by *. (C) Fibrosis develops in livers of mice serially injected with ConA. Livers isolated 24 h after the 8th weekly injection of ConA (*n* = 8; 12 mg/kg) or with saline (*n* = 4) in male mice before whole liver sections were stained with Trichrome with the sections then imaged at 4× to visualize blue collagen fibers. (D) Bridging fibrosis develops in livers of mice serially exposed to ConA. Liver sections of mice serially injected with saline or ConA (12 mg/kg weekly for 8 weeks) were stained with picosirius red with the resulting figures presented at 10× magnification. Quantitation of images with ImageJ shows collagen fibril content was significantly (*p* = 0.0229 in an unpaired *T* test) increased in ConA‐exposed mice with * denoting *p* < 0.05. (E) Extravascular platelets are present throughout liver parenchyma. Sections from saline or ConA‐injected mice were stained with platelet specific anti‐CD61 (β3 integrin; gpIIIa) displayed by anucleate platelets using DAB deposition from the HPR‐conjugated antibody. These sections were counterstained with hematoxylin to identify (blue) nucleated cells in livers of mice subsequent to eight serial injections of saline or ConA. Unpaired T test showed hepatic platelets were present in both cohorts. *p* = 0.3517 and so the difference between control and ConA‐treated mice was not significant. (F) P‐selectin displayed by activated platelets accumulates adjacent to healthy hepatocytes in livers of ConA‐treated mice. Sections from saline or ConA‐injected mice were stained with anti‐P‐selectin (CD62P) displayed on the surface of activated anucleate platelets, with nucleated cells then stained with hematoxylin. Quantitation by Image J showed ConA significantly increased the abundance of activated resident platelets. *p* = 0.0395 that was denoted (*) as less than 0.05.

### 2.9 EV Preparation and Analysis

LX2 cells were cultured overnight in serum‐free MEM media at 37° with a 5% CO_2_ atmosphere. The conditioned media was collected and centrifuged for 20 min at 2000 × *g*, 4° and then the cleared media was concentrated by retention over a 3 kDa cut‐off filter by centrifugation at 3500 × *g*. EVs were prepared from this media by first clearing large particles by centrifugation (30 min, 10 000 × *g*, 4°) and then recovering microparticles by ultracentrifugation (100 000 × *g*, 18 h, 4°), in a Beckman SW41Ti rotor. The supernatant was collected as EV‐depleted conditioned media, while the pellet was washed by resuspension in Hank's Balanced Salt Solution (HBSS) and recentrifuged (100 000 × *g*, 5 h, 4°) before this pellet was resuspended in HBSS. For EV analysis, the washed EV pellet was resuspended in PBS with the size and concentration determined by ZetaView PMX‐230‐Z‐488/640‐TWIN Laser System (Zetaview) from PARTICLE METRIX (Ammersee, Germany). The software version was ZetaView 8.05.16 SP3 and PS100 beads were used for calibration. EVs were diluted with extra pure water (~1/5000) to attain between 50 and 200 detected particles per frame with the following measurement setting: 488 laser scatter mode; sensitivity 80; frame rate 30; shutter 100; minimum brightness 30; maximum area 1000; minimum area 10; trace length 15; nm/class 10; classes/decade 64.

### 2.10 Western Blotting

Washed platelets, iPSC‐derived cells, or LX‐2 cells were lysed in reducing SDS loading buffer containing Coomassie blue along with a protease inhibitor cocktail containing pepstatin (Thermo Scientific). The proteins were resolved by electrophoresis in SDS‐containing 4%–20% cross‐linked gels before the proteins were transferred to PVDF membranes. These were blocked in 5% milk for 1 h at room temperature, incubated overnight with the stated primary antibodies, and then extensively washed prior to being detected by an appropriate immunoreactive horseradish peroxidase‐conjugated secondary antibody by ECL chemiluminescence.

### 2.11 TGF‐β Measurement

HEK‐Blue TGF‐β cells (50 000 cells in 180 μL) were distributed into wells of a flat‐bottom 96‐well plate prior to the addition of 20 μL of material to be assayed. This was either varied amounts of supernatants obtained from the aggregation of platelets induced by HSC‐derived EV, by recombinant human TGF‐β as the positive control, or HBSS. These plates were incubated overnight at 37°C in an atmosphere containing 5% CO_2_ before 20 μL aliquots of these supernatants were pipetted into a fresh flat‐bottom 96‐well plate in triplicate before the assay was initiated with 180 μL of resuspended QUANTI‐Blue Solution. The plate was incubated at 37°C for 30 min before secreted embryonic alkaline phosphatase (SEAP) was quantitated at 640 nm.

### 2.12 Enzymatic Activity Assays

Renin peptidolytic activity was assessed using an internally quenched Abcam fluorogenic renin assay kit (ab138875). Aliquots (50 μL) of platelet supernatant, recombinant human renin provided with the kit, or HBSS were added to wells of a Corning black 96‐well plate with a clear bottom. The assay was initiated by adding 50 μL of Renin Red Substrate solution to each sample. Fluorescence intensity was monitored in a Molecular Devices SpectraMAX i3x fluorescence plate reader at an emission wavelength of 590 nm after excitation at 540 nm. Data were recorded every 5 min for 60 min, with the slope converted to protein concentration in comparison to the linear recombinant renin standard, although, as noted by the manufacturer, the precise relative specific activity of the supplied recombinant renin standard was undefined. Thrombin enzymatic activity was assessed with the fluorogenic Thrombin Substrate III (Sigma 605211) by incubating 50 μL of platelet supernatant or enzymatically active α‐thrombin with 50 μL of thrombin substrate (1 mM in HBSS) in wells of a clear bottom black 96‐well microtiter dish. Emission intensity at 450 nm after excitation at 370 nm was measured every 5 min over 60 min using a Molecular Devices SpectraMAX i3x fluorescence plate reader.

### 2.13 Prorenin Knockdown

Prorenin was suppressed in LX‐2 cells by plating LX‐2 cells in 6 well plates (10^6^/well) one day prior to transfection and then replacing the medium with 1 mL of complete MEM 30 min prior to transfection. Stock solutions of Renin siRNA (ORIGENE Technologies SR 304032) and universal control siTran (2.0 siRNA) transfection reagents were made to 5 μM that was diluted 45× with the supplied transfection buffer before addition of siTran 2.0 reagent and addition to the target cells prior to incubation at 37° for 18 h. This media was replaced with serum‐free MEM and again cultured overnight before this media was replaced and the cells were again incubated overnight before this medium was collected, cleared by centrifugation at 10 000 × *g* for 30 min at 4°, and concentrated by filtration with a 3.0 Centricon. Nanoparticle analysis shows either transfection reagent led to secretion of equivalent numbers of EV.

### 2.14 Expression of Data and Statistics

Experiments were performed at least thrice using cells from different donors, a key control since platelet reactivity to all stimuli significantly varies among donors. The standard errors of the mean from all experiments are presented as error bars. Figures and statistical analyses were generated with Prism10 (GraphPad Software; RRID:SCR_002798). A value of *p* ≤ 0.05 was considered statistically significant and marked with a single asterisk, with *p* ≤ 0.01, *p* ≤ 0.001, 0.0001 marked by two, three, or four asterisks, respectively.

## 3 Results

### 3.1 Serial ConA Exposure Stimulates Murine Hepatic Fibrosis and Activation of Resident Intrahepatic Platelets

Hepatotoxic agents such as acetaminophen or CCl_4_ initially damage hepatocytes that then activate HSC, but the nature of the signals from damaged hepatocytes that actually activate fibrotic HSC to orchestrate the subsequent immune reaction [49] and liver damage is opaque. In contrast, ConA is a direct HSC TLR2 agonist [50] that induces murine hepatitis. Accordingly, we found ConA rapidly stimulated AKT phosphorylation in cultured human LX‐2 HSC, confirming ConA is an immediate agonist of these profibrotic cells (Figure 1A). The initial rapid liver inflammation from ConA exposure is followed by fibrosis that develops after 6–8 weeks of ConA exposure [51, 52] where activated lymphocytes are the downstream effectors and not initiators of this fibrosis [46, 47, 53]. We found (Figure 1B) by quantitative RT‐PCR analysis of collagen‐1 transcripts in livers of ConA‐treated mice relative to saline‐injected mice that ΔΔCt increased from 0 ± 0.9 to 2.1 ± 1.2 (*p* = 0.0119). We find ConA exposure for 8 weeks also enhanced trichrome blue staining of collagenous connective tissue fibers in the liver of these mice (Figure 1C). Similarly, picosirius red staining to visualize and quantitate hepatic collagen fibril content shows serial ConA exposure progresses to bridging fibrosis with a significant (*p* = 0.0229) increase in collagen fibril deposition (Figure 1D). CD61 immunohistochemistry to localize sites of platelet‐specific gpIIb/gpIIIa (CD41, CD61) adhesion complexes shows platelets were equivalently present throughout liver parenchyma in both control animals and those treated serially with ConA (*p* = 0.3157) (Figure 1E). However, platelets in livers of ConA‐treated mice had been activated as their expression of P‐selectin, a surface marker of activated platelets, was increased (*p* = 0.0395) by serial ConA exposure (Figure 1F). This staining correlated to activated platelets as P‐selectin was both unassociated with and smaller than hematoxylin‐labeled hepatocytes. These observations show that hepatic fibrosis develops in response to serial ConA stimulation that includes intrahepatic platelet activation.

### 3.2 LX‐2 HSC Release Thrombogenic Activity Associated With Extracellular Vesicles

**FIGURE 2:**
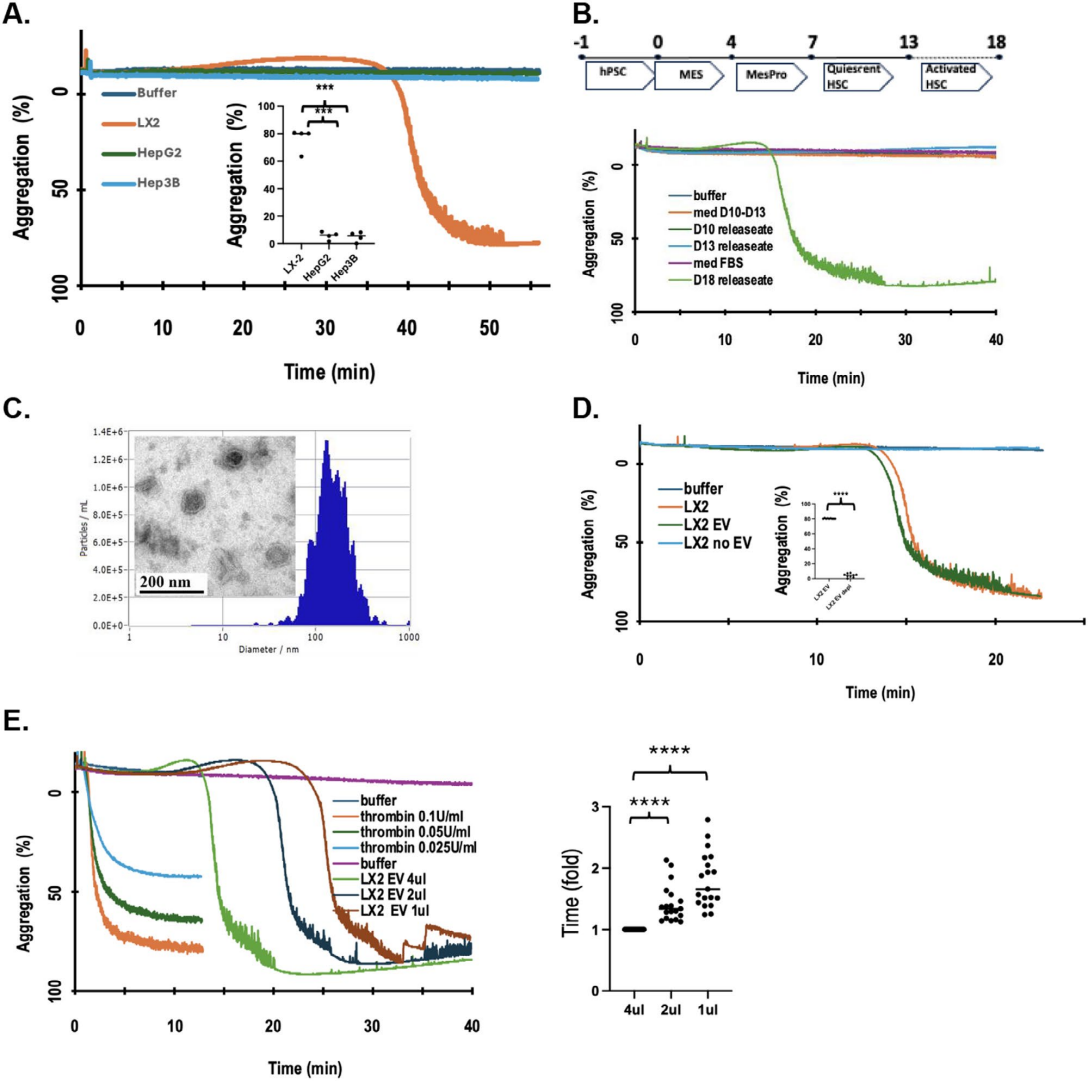
LX‐2 cell derived EV stimulate platelet activation. (A) Human platelets are stimulated by material(s) released from cultured LX‐2 HSC, but not from hepatoma cell lines. Serum‐free media was conditioned overnight by human HSC or hepatocyte cell lines, concentrated 10‐fold over 3 kDa Centricon cutoff filters, and then diluted 10× by injection into freshly isolated and washed human platelets stirring in an aggregometer cuvette. Light transmittance from opalescent washed platelet suspension (=0% aggregation) increases as platelet aggregation decreases scatter with platelet‐free plasma defined as 100% clearing. *N* = 4 individual blood donor biologic replicates with ***p < 0.001 for each comparison. (B) Media conditioned by initiated HSC matured in culture from human pluripotent stem cells stimulates platelet aggregation. *Upper temporal* schematic of human iPSC differentiation to mesenchymal cells, to mesenchymal progenitors, to quiescent HSC, and then to activated, but not stimulated, HSC. Media from these cultures on the stated days/state of maturation were assayed for platelet stimulation by aggregometry. *N* = 6 for day 18 and media, and *N* = 3 culture for day 10 and 13. Controls include buffer with or without FBS, or cell‐free growth media used during days 10–13. (C) LX‐2 cell conditioned media contains extracellular vesicles. LX‐2 cell media was concentrated by ultracentrifugation to recover particulate material before the particles were resuspended and diluted 5000‐fold to enable particle number and size assessment by Nanoparticle Tracking Analysis. *N* = 3 An aliquot of the resuspended pellet was diluted, fixed, and stained for transmission electron microscopy (inset) *N* = 3. (D) LX‐2 cell EV exclusively express platelet stimulatory activity. Growth media conditioned overnight by LX‐2 cells was cleared of cellular material and debris by low speed centrifugation before membranous vesicles were isolated by high speed centrifugation (100 000 × *g*). The pellet recovered from this centrifugation was reconstituted in a volume of HBSS buffer equivalent to the original volume and then this or the EV‐depleted media was tested for platelet aggregatory activity. *N* = 9 biologic replicates. ****p < 0.0001 (E) EV abundance alters the length of hysteresis prior to initiation of aggregation, but not the extent of aggregation in response to HSC‐EV. Aggregometry of washed human platelets responding to varied concentrations of active alpha thrombin (*N* = 3 biologic replicates) or in response to increasing amounts of LX‐2 conditioned media. *N* = 19 biologic replicates. *Right*. Purified EV induce concentration‐dependent platelet aggregation. ****p < 0.0001 by one way Anova.

We tested the postulate that the human LX‐2 HSC line expresses platelet stimulatory activity by collecting serum‐free media after overnight incubation. We reduced the volume of this conditioned media 10‐fold by ultrafiltration, and then diluted this concentrate 10‐fold by injection into freshly isolated human platelets being stirred in cuvettes in the standard aggregometry assay for platelet activation. Media conditioned by LX‐2 cells induced an early slight increase in light transmittance, reflecting either a change in platelet shape [54] and/or formation of transient microaggregates [55], that was immediately followed by robust aggregation presenting as a rapid decrease in opacity of the turbid platelet suspension as activated platelets coalesced (Figure 2A). In contrast, media similarly conditioned overnight by either human HepG2 or HepG3 hepatocyte cell lines failed to stimulate platelet activation and aggregation.

We question whether the release of stimulatory activity from LX‐2 cells was a selective property of the cultured LX‐2 cell line by differentiating human induced pluripotent stem cells (iPSC) first to mesenchymal cells [56], then to quiescent HSC, and finally to activated, but unstimulated, HSC. Mesenchymal cells did not release platelet agonistic activity, nor did their differentiation to quiescent HSC result in the release of platelet agonists (Figure 2B). However, after maturation, these cells resulted in the release of material(s) that stimulated platelet aggregation. Moreover, the response of platelets to this iPSC‐derived material displayed the same, novel delayed temporal response pattern as the material released from LX‐2 cells.

Platelets are stimulated by just a few soluble agonists, for example, thrombin, thromboxane A_2_, ADP, serotonin, or Platelet‐activating Factor, through their cognate platelet G protein receptors [57, 58]. The stimulatory agent(s) recovered from LX‐2 cell conditioned media, however, were larger than these soluble platelet agonists as activity was retained by a 3 kDa cutoff filter. The resulting retentate containing larger proteins, and potentially thrombin, additionally would include LX‐2 cell membrane fragments, EV, and exosomes. We separated these membranous components by ultracentrifugation since HSC‐derived extracellular vesicles (EV) transport a host of regulatory RNAs and protein mediators [59] targeting epithelium, endothelium, and innate immune cells to promote hepatic fibrosis [60, 61]. We resuspended the recovered pelleted material in an equivalent volume of buffer to find the reconstituted material contained a single population of 2.2 × 10^12^ particles/ml centered about a size of 96 ± 64 nm by nanoparticle tracking analysis (Figure 2C). This size is consistent with EV, and imaging these particles by transmission electron microscopy revealed particles with the sealed bilayer membranes characteristic of EV (Figure 2C inset). We separately tested EV recovered by ultracentrifugation and their corresponding EV‐free media as platelet agonists to find that EV were as effective as the unfractionated conditioned media, while the EV‐depleted media lacked stimulatory activity (Figure 2D).

The delay in the response of platelets to HSC‐derived EV was remarkable as platelets carried in flowing blood must instantly respond as they pass sites of intravascular damage, exemplified by their immediate response to thrombin stimulation (Figure 2E). In addition, the response of platelets to soluble agonists like thrombin is graded with increasing amounts of agonist increasing the numbers of responding cells that progressively increase the extent of aggregation of the population. In contrast, the response of platelets to LX‐2 or iPSC‐HSC EV was characterized by a profound delay with activation, once initiated, always complete no matter the amount of stimuli. Moreover, the time to initiation of aggregation varied with the number of inciting EV, and not the number of responding cells, where a fourfold reduction in EV concentration significantly (*p* < 0.0001) delayed the time to half maximal activation by nearly twofold. HSC therefore release an atypical, EV‐associated platelet stimulatory activity.

### 3.3 Activation of LX‐2 EV Associated Prorenin Promotes Platelet Stimulation

**FIGURE 3:**
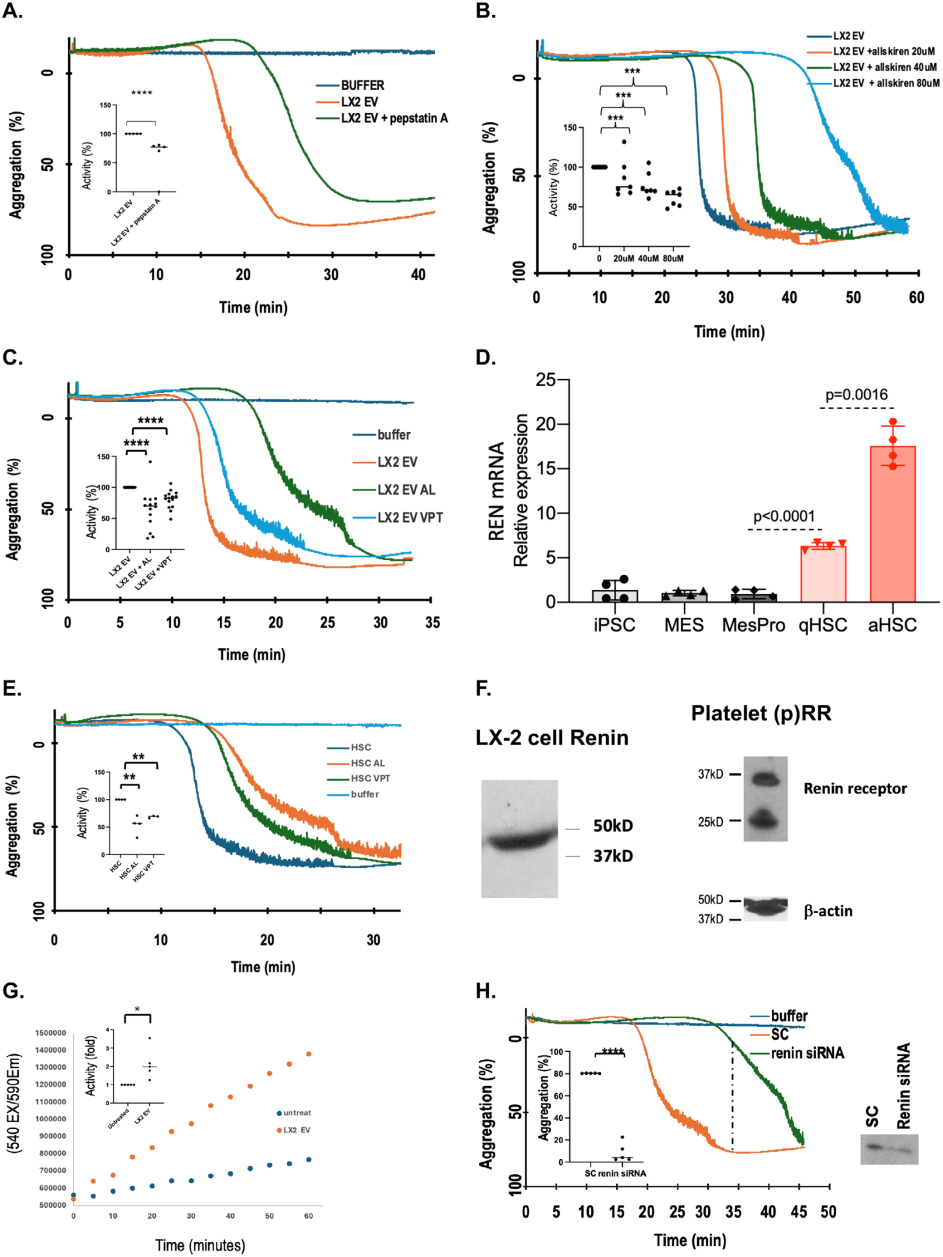
LX‐2 derived EV contain renin activity that stimulates delayed platelet activation. (A) The aspartyl protease inhibitor pepstatin suppresses LX2 induced platelet activation. LX‐2 cell EV were preincubated with pepstatin (10 μg/mL, 10 min), or not, prior to assessing platelet activation induced by LX‐2 EV. HBSS buffer the EV were resuspended in was the negative control. ***p < 0.001 unpaired *T* test, *N* = 5 biologic replicates. (B) Renin enzymatic activity underlies platelet stimulation by LX‐2 EV. Freshly isolated human platelets were incubated (10 min, 1000 rpm) in aggregometer cuvettes with the stated concentrations of the DRI Aliskiren. Aggregation was then initiated by the addition of LX‐2 EV at *t* = 0. *N* = 7 biologic replicates. ***p < 0.001 by one way ANOVA. (C) The Direct Renin Inhibitor VTP23999 and Aliskiren inhibit HSC EV‐induced platelet aggregation. Platelets stirring in aggregometer cuvettes were pretreated, or not, with VTP23999 (40 μM) or Aliskiren (40 μM) prior to the addition of buffer or washed EV recovered from LX‐2 cell conditioned media. VTP23999 *N* = 15 biologic replicates ****p < 0.0001; Aliskiren *N* = 14 biologic replicates unpaired *T* test *p* < 0.0001. (D) Renin mRNA accumulates during iPSC maturation to initiate HSC. Transcript levels of the *REN* gene quantified by RT‐qPCR were assessed as human iPSC cells were differentiated to quiescent and then initiated HSC. mean ± SD using a two‐tailed unpaired *t*‐test with Welch's corrections to calculate exact *p*‐values from 4 independent replicates. (E) Renin enzymatic activity promotes platelet aggregation in response to EV from iPSC‐derived initiated HSC. Platelet aggregation induced by media conditioned by iPSC matured to initiated HSC in the presence or absence of 40 μM Aliskiren (*N* = 4 biologic replicates) or VTP23999 (*N* = 3 biologic replicates) **p < 0.01 with *p* = 0.0012. (F) LX‐2 cell derived EV contain prorenin while platelets abundantly express the prorenin receptor (p)RR. (*left*) Western blot for renin (37.5 kDa) and prorenin (~42–45 kDa) in EV isolated by ultracentrifugation from LX‐2 cell conditioned media, and (*right*) prorenin receptor (apparent ~42 kDa mobility) expressed by LX‐2 cells or washed human platelets. (G) The combination of platelets incubated with LX‐2 EV develops renin enzymatic activity. Renin enzymatic activity was assayed using a fluorogenic TF3/TQ3‐labeled peptide substrate (Abcam) to assess fluorescent resonant energy transfer over time as platelets were incubated with or without purified LX‐2 EV. *N* = 5 assay replicates. Unpaired *T* test *p* = 0.0172. (H) Renin knockdown in LX‐2 cells reduces EV‐associated renin and suppresses LX‐2 EV‐induced platelet aggregation. LX‐2 cells were incubated overnight with REN siRNA or a universal scrambled control siRNAs, the transfection media was removed and the cells were incubated with complete MEM overnight, this was removed and discarded and the cells incubated overnight for a second time before this conditioned media was collected, cleared by centrifugation, and concentrated by Centricon filtration. EV from knockdown or scrambled control cells were added to stirring platelets at *t* = 0 with aggregation assessed by clearing as before. The amount of aggregation after 35 min of incubation was quantitated with REN siRNA EV significantly less effective than EV from scrambled control‐treated cells. *N* = 5 biologic replicates ****p < 0.0001.

We sought to identify the relevant EV‐associated agonist, and initially considered that the prolonged delay prior to platelet activation was consistent with a time‐dependent proteolytic conversion of an inactive zymogen to a stimulatory protease. To test this, we incubated LX‐2 cell conditioned media with a cocktail of protease inhibitors to find one cocktail was without effect, but a cocktail from a second manufacturer abolished platelet activation (not shown). The difference between the two cocktails was the additional presence of pepstatin in the effective cocktail, and pepstatin itself delayed, and so significantly (*p* < 0.0001) inhibited (shown by its concentration‐response relationship in Figure 2E) platelet aggregation induced by LX‐2 cell EV (Figure 3A). Pepstatin is a highly selective inhibitor of aspartyl proteases, but the only human member of this very small family that has any activity at the neutral pH [62] of our assays, and is inhibited by pepstatin [63], is renin. Renin is specifically inhibited by the designer drug Aliskiren [31] and this FDA‐approved, First‐in‐Class DRI suppressed platelet aggregation (*p* < 0.001) induced by LX‐2 EV in a concentration‐dependent fashion (Figure 3B). The more recently developed DRI VTP23999 [64] also inhibited (*p* < 0.0001) platelet aggregation by LX‐2 EV (Figure 3C).

HSC isolated from patients with cirrhotic liver disease express renin, but unactivated HSC of normal individuals do not [29]. We assayed REN gene transcription by qPCR as human iPSC matured to mesenchymal cells, then to quiescent, and finally to activated HSC. We found renin expression in mesenchymal cells did not differ from the starting iPSC, but that further differentiation to quiescent and then activated cells increased REN transcript levels by 5‐ and 17‐fold, respectively (Figure 3D). These REN transcripts directed protein synthesis as EV released from these iPSC‐derived HSC stimulated platelets in both an Aliskiren‐inhibitable and VTP23999‐inhibitable fashion (*p* < 0.01) (Figure 3E). Western blotting for the presence of renin in LX‐2 cell derived EV revealed a single immunoreactive band, but the apparent size of this material at ~42 to 45 kDa shows this protein was prorenin and not mature 37 kDa renin (Figure 3F *Left*). Prorenin is enzymatically inactive, but both LX‐2 cells [41] and human platelets (Figure 3F *Right*) express the membrane‐associated prorenin receptor (p)RR that nonproteolytically generates renin enzymatic activity by the prorenin zymogen after (p)RR ligation [43]. We used a fluorogenic renin peptidase assay to establish that the combination of HSC‐EV and platelets interacted to generate renin peptidolytic activity that exceeded that displayed by platelets alone (Figure 3G). We knocked prorenin expression down in LX‐2 cells by overnight incubation with REN siRNA followed by two 24 h incubations with fresh medium prior to collecting the EV these cultures released. The REN siRNA knockdown reduced renin immunoreactivity in the EV they released relative to cells treated with its scrambled control (Figure 3H inset) and significantly (*p* < 0.001) reduced platelet aggregation induced by these EVs relative to those treated with buffer or a universal scrambled control siRNA (Figure 3H).

### 3.4 EV‐Associated Prorenin Is Glycosylated With Deglycosylation Mitigating Platelet Stimulation

**FIGURE 4:**
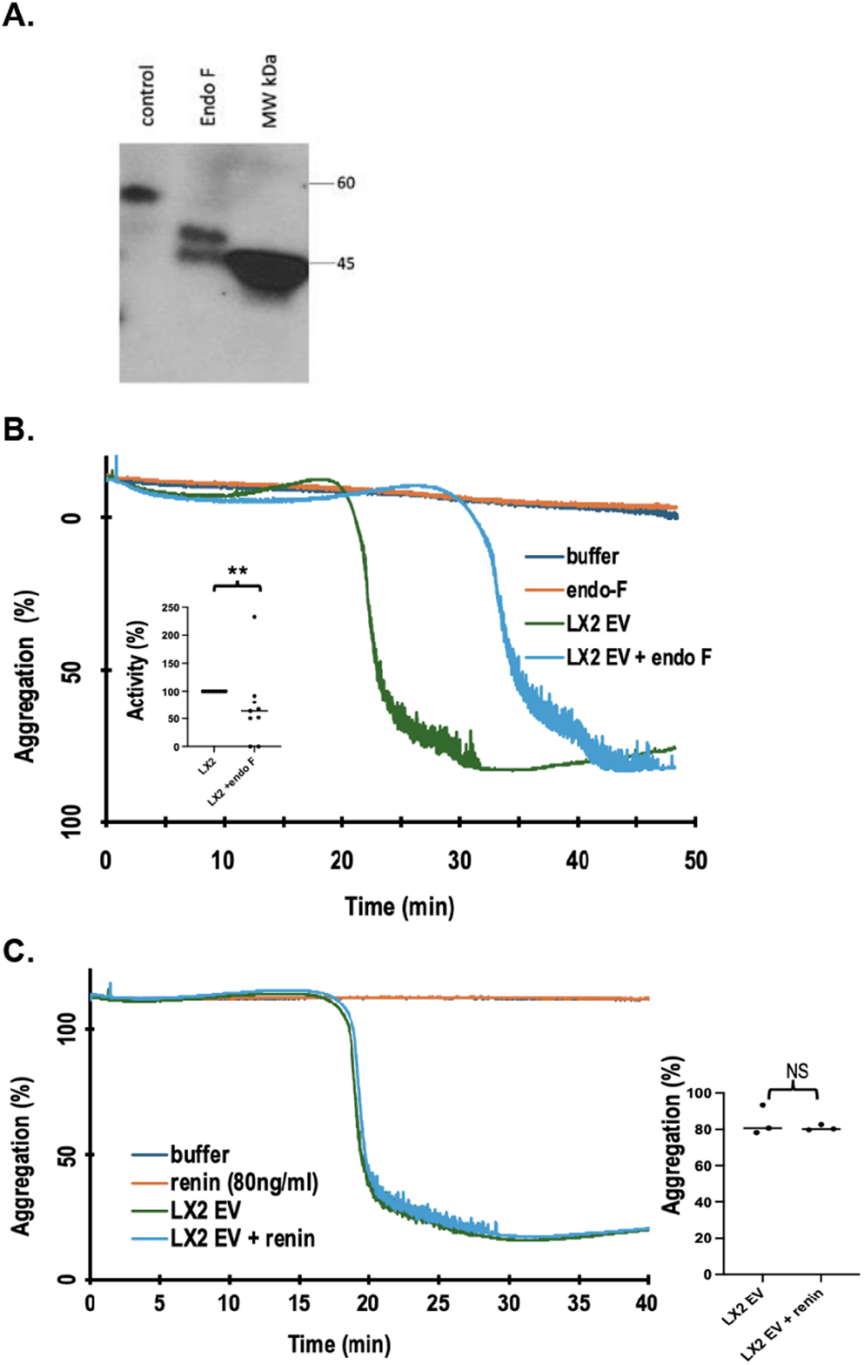
LX‐2 cell EV present glycosylated prorenin to platelets to stimulate aggregation. (A) LX‐2 HSC‐EV prorenin is accessible to soluble deglycosylase EndoF. EV isolated from LX‐2 cells were pre‐treated, or not, (50 000 U, 3 h) with EndoF before the sample was denatured, resolved by electrophoresis before the gel was immunoblotted with anti‐renin or anti‐ovalbumin antibody. (B) EndoF pre‐treatment inhibits LX‐2 EV‐induced platelet aggregation. LX‐2 EV were pre‐treated, or not, with EndoF as in the preceding panel prior to addition to platelets stirring in aggregometer cuvettes. As a control for EndoF carryover, platelets alone were treated with EndoF alone during aggregometry *N* = 9, *p* = 0.0019 depicted as ** for *p* < 0.01. (C) Soluble renin is not a platelet agonist. Human platelets stirring in aggregometer cuvettes were stimulated with either 80 ng/mL recombinant renin, purified LX‐2 cell EV, the combination of recombinant renin and LX‐2 cell EV, or HBSS buffer. *N* = 3 platelet donors with outcomes depicted in the right panel. *p* = 0.533.

EV contain intravesicular, transmembrane, and surface‐associated proteins, but if prorenin is an internal cargo, then platelet stimulation likely would follow internalization or fusion rather than interacting with an external platelet receptor like (p)RR. Renin and prorenin are glycosylated and ligands for the lectin ConA [65], so we determined whether prorenin associated with HSC‐EV was externally accessible by treating LX‐2 cell EV with peptide: N‐glycosidase F (EndoF). Digestion by this soluble enzyme reduced the apparent electrophoretic molecular weight of all vesicle‐associated prorenin (Figure 4A), so all EV‐associated prorenin was externally available. Pretreating LX‐2 EV with EndoF reduced subsequent EV‐stimulated platelet aggregation *p* < 0.01 (Figure 4B). In contrast, active unglycosylated recombinant renin was ineffective as a platelet agonist (Figure 4C). Additionally, soluble renin alone failed to augment LX‐2 cell EV‐induced aggregation. These data establish that EV‐associated prorenin is available as a platelet agonist when displayed by HSC‐EV and suggest its presentation, at least in part, derives from its glycosylation.

### 3.5 HSC EV Interact With Platelets to Initiate the Extrinsic Coagulation Cascade That Generates Platelet‐Stimulatory Thrombin

**FIGURE 5:**
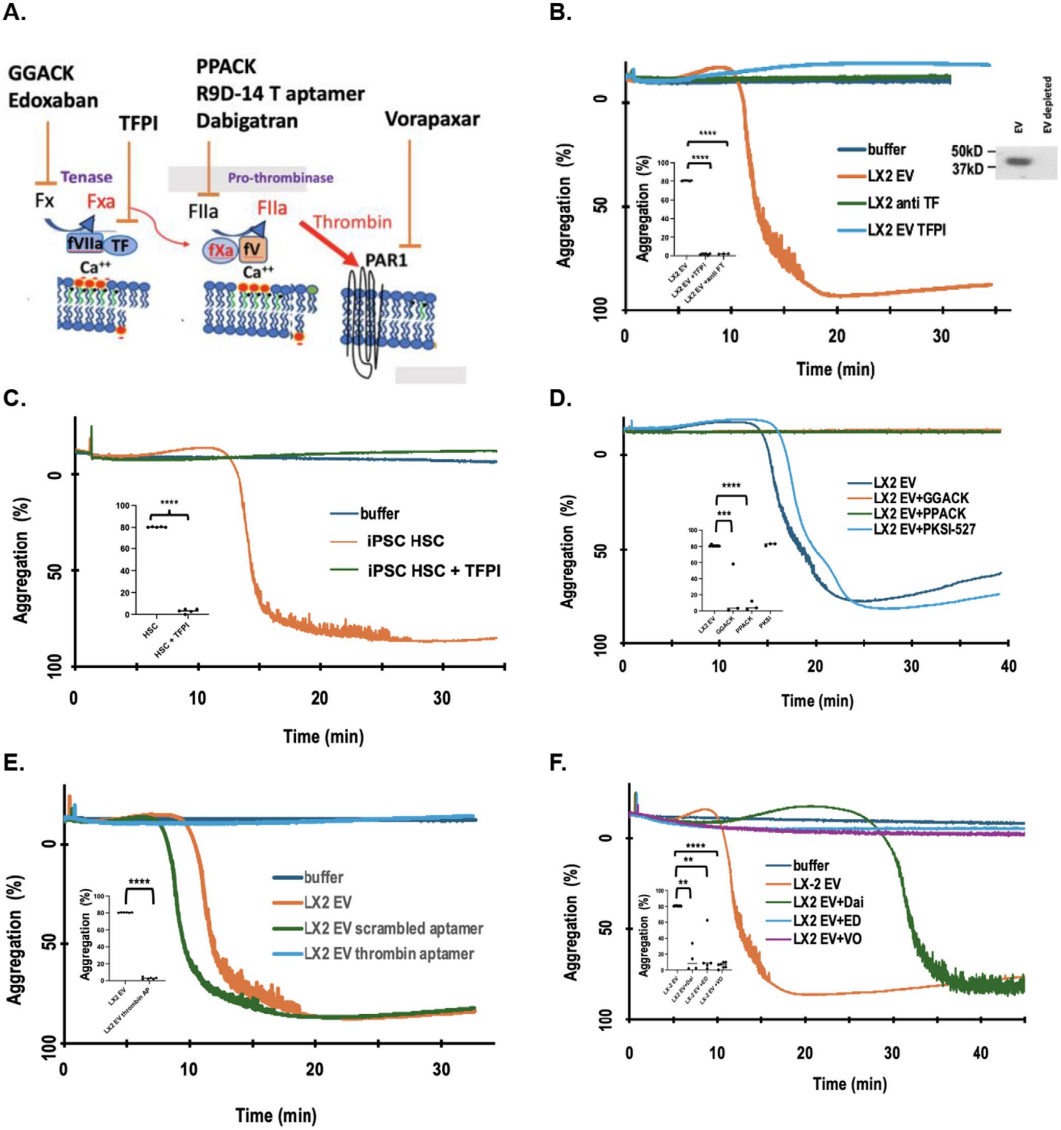
LX‐2 derived EV interact with platelets to initiate the extrinsic and common coagulation cascade to generate thrombin that stimulates the platelet PAR1 thrombin receptor. (A) Sites of inhibition by specific inhibitors or FDA‐approved clinical inhibitors of the extrinsic and common coagulation complexes. Coagulation complexes form on the surface of activated platelets where the extrinsic coagulation cascade is initiated by activated Tissue Factor (Factor III; CD142) complexed with Factor VIIa that proteolyzes inactive Factor X to active Factor Xa in an extrinsic tenase complex. This complex, and the prothrombinase complex, forms by ligation of their Gla residues to Ca^++^ bridging to anionic phosphatidylserine displayed on the surface of activated platelets. Proteolysis of prothrombin to active thrombin by active Factors Xa and Va generates thrombin, the stimulating ligand for the Protease‐Activated Receptor‐1 PAR1. (B) LX‐2 EV induce Tissue Factor‐dependent platelet activation. Platelet aggregation incubated by LX‐2 cell conditioned media in the absence or presence of Tissue Factor Pathway Inhibitor (TFPI), or by anti‐TFPI. *N* = 5 biologic replicates ****p < 0.0001. *Inset*: Tissue Factor western blot of purified LX‐2 cell EV or their EV‐depleted media. (C) Stimulation of platelet aggregation by iPSC‐derived initiated HSC requires Tissue Factor activity. Platelet aggregation in response to conditioned media from activated (day 18) iPSC‐derived HSC stimulated platelet aggregation that was abolished by the presence of TFPI. *N* = 5 biologic replicates ****p < 0.0001. (D) The enzymatic activity of thrombin and Factor Xa is necessary for LX‐2 EV‐induced platelet activation. Aggregation induced by LX‐2 cell conditioned media was abolished by irreversible thrombin inhibition by PPAK (Phe‐Pro‐Arg‐chloromethyl ketone) peptide or Factor Xa inhibition by the irreversible GGACK peptide. Inhibition of plasma kallikrein (PKSI‐527) of the intrinsic coagulation cascade was without effect. *N* = 3 biologic replicates. ***p < 0.001, ****p < 0.0001. (E) Thrombin inhibition by the RNA aptamer R9D‐14T abolished platelet aggregation in response to EV purified from LX‐2 cell conditioned media. Platelets were treated with 200 nM of the R9D‐14T aptamer selected to inhibit thrombin enzymatic activity, or a scrambled RNA control, for 10 min prior to addition of either buffer or LX‐2 cell derived EV. *N* = 4 biologic replicates ****p < 0.0001. (F) Pharmacologic inhibition of thrombin formation and signaling suppresses LX‐2 cell EV induction of platelet aggregation. Platelet aggregation in response to buffer or material released overnight from LX‐2 cells in the presence or absence of the FDA approved Edoxaban (ED; *N* = 5) inhibitor of Factor Xa activity, Dabigatran (Dai; *N* = 4) inhibitor of thrombin activity, or Vorapaxar (VO; *N* = 5) inhibitor of the PAR‐1 thrombin receptor. **p < 0.01, ****p < 0.0001.

We sought to mechanistically understand how HSC‐EV activate platelets and considered that platelets respond to just a few mediators and, of these, only thrombin can activate all platelets [20]. Prothrombin circulates as an inactive zymogen that is activated through the common coagulation cascade where inactive Factor X is activated in a “tenase” complex that forms Factor Xa that then acts in a prothrombinase complex to proteolyze prothrombin to active thrombin (Figure 5A). This common coagulation cascade is stringently regulated either by the upstream Tissue Factor–dependent extrinsic cascade or the contact–dependent intrinsic coagulation cascade [18]. Western blotting showed that LX‐2 EV express Tissue Factor (Figure 5B *inset*), and platelet aggregation induced by LX‐2 cell EV was abolished (*p* < 0.0001) by Tissue Factor Pathway Inhibitor (TFPI) or by inhibitory anti‐Tissue Factor antibody (Figure 5B). Similarly, platelet activation by iPSC‐derived HSC was abolished by TFPI *p* < 0.0001 (Figure 5C). In contrast, the inclusion of the kallikrein inhibitor PKSI 527 that suppresses the intrinsic coagulation cascade did not significantly affect LX‐2 EV induced aggregation (Figure 5D).

We determined whether the enzymes in the common coagulation pathway downstream of the extrinsic coagulation cascade were necessary for platelet stimulation by LX‐2 cell EV to find that the irreversible Factor Xa inhibitor glutamyl‐glycyl‐arginyl chloromethylketone (GGACK) abolished platelet aggregation *p* < 0.001 (Figure 5D). Similarly, irreversible inhibition of thrombin enzymatic activity with phenyl‐prolyl‐arginyl chloromethylketone (FPRCK or PPACK) also abolished platelet aggregation *p* < 0.0001. Supporting the essential role of thrombin, we found the RNA aptamer R9D‐14T selected to specifically block thrombin enzymatic activity [66] abolished platelet aggregation in response to LX‐2 cell EV *p* < 0.0001 (Figure 5E). Finally, we tested clinically employed pharmacologic inhibitors to find that Edoxaban, an inhibitor of active Factor Xa, and the thrombin inhibitor Dabigatran abolished platelet aggregation *p* < 0.01 (Figure 5F). The primary signaling receptor expressed by human platelets for thrombin is protease‐activated receptor‐1 (PAR1), and its pharmacologic inhibition by Vorapaxar also abolished platelet activation *p* < 0.0001. These data establish platelet activation by HSC‐derived EV proceeds through the common and extrinsic coagulation cascades to generate the PAR‐1 agonist thrombin.

### 3.6 Hysteresis in Prothrombin Proteolysis and Activation Corresponds to Delayed LX‐2 EV Induced Platelet Activation

**FIGURE 6:**
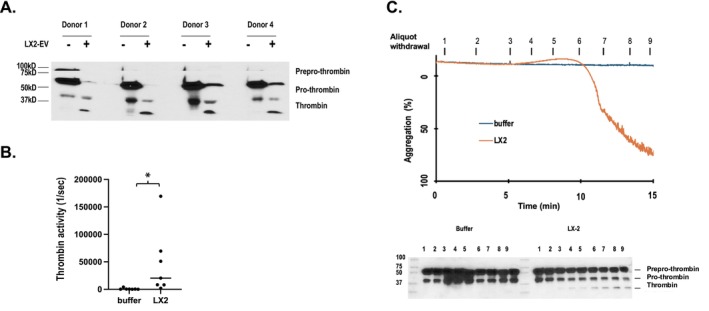
Delayed thrombin formation correlates to delayed platelet activation. (A) Inactive thrombin isoforms are proteolyzed as platelets interact with LX‐2 derived EV. Western blotting of thrombin isoforms after platelet aggregation induced by LX‐2 cell derived EV. *N* = 4 blood donors. (B) Development of thrombin enzymatic activity during co‐incubation of platelets with LX‐2 cell conditioned media or buffer. Thrombin activity was assayed with the thrombin specific fluorogenic substrate benzoyl‐FVR‐AMC after platelets were incubated for 30 min with buffer or LX‐2 conditioned media. *N* = 7, *p* = 0.012, *p < 0.05 (C). Time‐dependent proteolysis of thrombin zymogens to mature thrombin as platelets aggregated in response to LX‐2 EV. *Top*: Temporal sampling scheme as platelets changed shape and aggregated in response to LX‐2 conditioned media. Samples collected from the foregoing assay at the stated times prior to initial change in platelet shape (samples 1–3), as platelets changed shape (samples 4–6), or at subsequent times as platelets aggregated (samples 7–9) and the opacity of the suspension cleared. *Bottom*: Thrombin isoform western blotting of aliquot samples. *N* = 3.

Delayed platelet activation is a non‐canonical response, but understanding that EV‐induced aggregation depends on thrombin formation and its signaling to PAR1 indicates this delay in aggregation must reflect a delay in the generation of active thrombin. To test this, we first confirmed that platelets interacting with HSC EV proteolyzed thrombin zymogens to active alpha thrombin by collecting media after platelets were incubated with HSC‐derived EV or buffer alone. This showed material from all blood donors contained prepro‐ and pro‐thrombin zymogens in the absence of active alpha thrombin, while incubation with LX‐2 EV reduced the abundance of thrombin zymogens with the appearance of proteolytically processed thrombin (Figure 6A). We confirmed this thrombin was active by assessing cleavage of the fluorogenic thrombin peptide substrate BOC‐quenched valyl‐proylyl‐arginyl‐aminomethylcomarin. This fluorogenic assay showed platelets incubated with LX‐2 cell EV for 30 min generated robust thrombin enzymatic activity (Figure 6B). Platelet aggregation induced by LX‐2 cell EV was notable for the hysteresis prior to activation, so to determine whether the time‐dependent appearance of thrombin correlated to the unusual temporal pattern of platelet aggregation induced by LX‐2 cell EV, we collected sequential aliquots at the times marked in Figure 6C from the aggregometer cuvettes containing platelets interacting with LX‐2 cell EV. Western blotting for thrombin isoforms in these aliquots revealed both prepro‐thrombin and pro‐thrombin zymogens in the initial aliquots, while proteolytically processed thrombin began to appear in aliquots 4 and 5 as platelets began to change shape and then increased as platelets began to aggregate. In contrast, platelets incubated in buffer alone failed to proteolyze prothrombin zymogens, so the novel delay in platelet response to HSC EV reflects a delay in the formation of active thrombin.

### 3.7 Platelets Release TGF‐β in Response to LX‐2 Cell EV That Stimulates LX‐2 Cell Activation and Matrix Deposition in Naïve LX‐2 Cells

**FIGURE 7:**
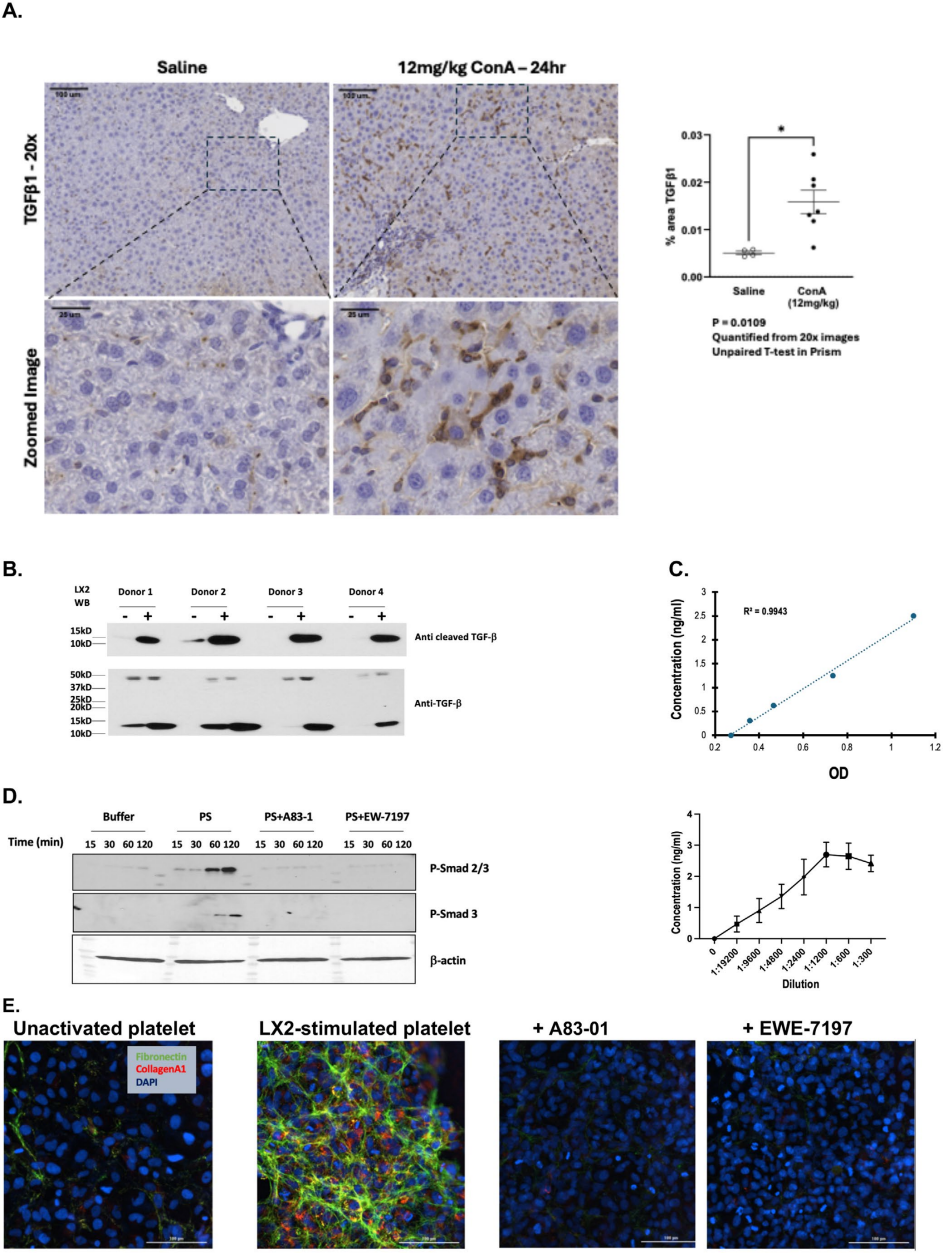
LX‐2 activated platelets release TGF‐β that stimulates LX‐2 cell matrix deposition. (A) Punctate, anuclear sites of TGF‐β expression increases in livers of mice serially exposed to ConA. Sections from saline or ConA‐injected mice were stained with anti‐TGF‐β using DAB deposition from the HRP‐conjugated antibody. These sections were counterstained with hematoxylin (blue) to identify nucleated cells. Unpaired *T* test *p* = 0.019. *p < 0.05 (B) Platelets stimulated with LX2 EV shed proteolyzed TGF‐β. Freshly isolated human platelets from four donors were treated with buffer, or thrombin as a positive control, or with LX‐2 cell conditioned media for 30 min. Proteins in this media were separated by reducing SDS‐PAGE and blotted with anti‐TGF‐β that detects only its proteolyzed active form or with antibody that recognizes both nascent and cleaved TGF‐β. (C) TGF‐β released by platelets incubated responding to LX‐2 EV is biologically active. (*Top*) The concentration‐response relationship of TGF‐β and secreted alkaline phosphatase assay from HEK‐Blue cells is linear. HEK‐Blue reporter cells were treated with the stated amounts of recombinant human TGF‐β and incubated overnight before the conditioned media was assessed with QUANTI‐blue solution at 640 nm for secreted alkaline phosphatase activity. (*Bottom*) Assessment of bioactive TGF‐β released by LX‐EV stimulated platelets. Overnight supernatants from the interaction of platelets with LX‐2 cell conditioned media were cleared by centrifugation and then diluted into growth media of HEK293 Blue reporter cells. After overnight incubation at 37° under 5% CO_2_, the resulting media was assayed with QUANTI‐blue as before. *N* = 3 biologic samples. (D) LX‐2 EV stimulated platelets release TGF‐β that stimulates Smad transcription factor phosphorylation in naïve LX‐2 cells through TGFbR1. Naïve LX‐2 cells were treated as a function of time with buffer, activatd platelet supernatants (PS) from platelet/LX‐2 cells co‐cultured for the stated times, or this media containing either A83‐01 or EW‐7197 (either at 100 nM) to inhibit TGFbR1. The target LX‐2 cells were washed, lysed and their proteins resolved by reducing SDS‐PAGE prior to immunoblotting with antibodies ligating either phospho‐Smad 2/3 or phospho‐Smad3. (E) TGF‐β activity released by platelets activated with LX‐2 EV promotes LX‐2 fibronectin and collagen deposition by naïve LX‐2 cells. Media from the co‐incubation of platelets with HSC‐EV as in the preceding panel was incubated with naïve LX‐2 cells for 48 h in the presence or absence of either of the two TGFbR1 inhibitors (100 nM) before the media was removed, the plate washed and then fixed with paraformaldehyde. Pro‐fibrotic collagen (red) and fibronectin (green) were visualized by two color immunofluorescence after DAPI staining nuclei blue. *N* = 3 replicates.

Activated platelets, in contrast to quiescent cells, are a primary source of soluble pro‐fibrotic TGF‐β [67]. Immunohistochemistry showed the livers of saline injected control mice contain little TGF‐β, presenting as occasional punctate accumulations that were not associated with DAPI‐staining nucleated cells (Figure 7A). In contrast, livers of ConA‐treated mice displayed a significant (*p* = 0.0109) increase in TGF‐β expression where TGF‐β inclusions were both more numerous and more dispersed than in control animals. This TGF‐β still appeared as punctate inclusions and still were poorly associated with DAPI‐staining hepatocytes that remained intact and healthy during the serial exposure to ConA. In vitro analysis confirmed that quiescent platelets released little proteolyzed TGF‐β, while media from platelets co‐incubated with LX‐2 cell EV released soluble proteolytically processed TGF‐β (Figure 7B). This soluble TGF‐β was active as it strongly stimulated TGF‐β transcription in HEK 293 cells that express a secreted alkaline reporter under the control of Smad transcription (Figure 7C). Comparison to the kit's recombinant control showed platelets stimulated by LX‐2 cell EV released the equivalent of microgram quantities of TGF‐β. Platelet‐derived TGF‐β was an effective agonist for LX‐2 cells since it stimulated phosphorylation of Smad2 and Smad3 transcription factors in naïve LX‐2 cells, with this response being blocked by inhibition of TGFbR1 by either A83‐01 [68] or EW‐7197 (Vactosertib) [69] (Figure 6D). Platelets activated by LX‐2 cell EV stimulated deposition of collagen A1 and fibronectin fibrils by naïve LX‐2 cells, with production of these matrix proteins also suppressed by A83‐01 or EW‐7197 (Figure 6E). We conclude activated platelets residing within murine liver parenchyma of ConA exposed mice can be a source of intrahepatic pro‐fibrotic TGF‐β.

## 4 Discussion

Hepatic fibrosis is modeled in mice through sequential exposure to hepatotoxic agents like CCl_4_ or acetaminophen, but how hepatocytes damaged by these agents then promote the transition of quiescent HSC to a profibrotic phenotype is obscure [70]. In contrast, the lectin ConA ligates and activates Tlr2 signaling [50, 71] in primary murine HSC, with mice serially exposed to ConA progressing from initial hepatitis to fibrosis [51, 52]. ConA orchestrates the response of multiple cells in liver injury [49] that stimulate the expression of γIFN [72] necessary for the subsequent T cell‐induced hepatic injury [49, 73, 74]. Reconstitution of hepatic injury by recombinant γINF in Rag2‐deficient mice [74] shows events prior to T cell activation precede ConA‐induced liver fibrosis. Intercellular HSC communication includes the EVs they release [60], and we can now include platelets as recipients of, and respondents to, this HSC EV communication axis.

Platelets normally reside within liver parenchyma (Figure 1), with inflammation increasing their numbers [5, 6], with activated platelets contributing to hepatic fibrosis [3, 16, 75, 76]. Liver parenchyma is a low‐flow neighborhood enabling prolonged interactions of platelets with small, diffusible HSC EV in a lymphatic environment containing reduced amounts of coagulation protease inhibitors [77]. We establish that platelets are activated over time by EV derived from activated HSC, yet the proteome of LX‐2 EV does not contain known platelet agonists [59], soluble platelet agonists were excluded by ultrafiltration of HSC conditioned media, and established platelet agonists are all immediately stimulatory. The basis for each of these differing responses is hysteresis in platelet‐dependent prothrombin activation. We found EV from LX‐2 cells or activated iPSC‐derived HSC interacted with platelets over time to form extrinsic tenase and prothrombinase coagulation complexes that generated thrombin, the single complete platelet agonist [20], and that the delay in the formation of enzymatically active thrombin correlated to hysteresis in the onset of platelet activation. Thrombin was the sole agent responsible for this platelet activation as aggregation was abolished by the complete loss of thrombin enzymatic activity by any of several specific agents, as well as by blockade of subsequent PAR1 signaling. Thrombin rapidly amplifies its own production, so incomplete inhibition of its activation merely delays initiation of the amplification phase of thrombin formation. Incomplete inhibition, then, presents as a longer hysteresis phase followed by complete aggregation of the entire platelet population once thrombin begins to induce its own formation. Platelet activation by HSC EV is thus exquisitely responsive to the rate of thrombin formation rather than a novel response of platelets to an unknown agonist.

We show prorenin mRNA is expressed by activated HSC and prorenin is released as a surface component of the EV they shed. Prorenin is not catalytically active as its pro domain obscures its active site, but platelets express (p)RR [78] that binds and induces a conformational alteration that non‐proteolytically activates renin catalytic activity by the ligated prorenin zymogen [45]. Accordingly, the DRI Aliskiren or VTP27999 [44, 79], along with the class‐specific inhibitor pepstatin, blocked platelet activation by HSC‐derived EV. Prorenin knockdown in LX‐2 cells by siRNA confirmed the role of this protein in LX‐2 EV induced platelet activation. This novel intercellular interaction to generate renin activity may be an attractive target to modulate as blockade or knockdown of (p)RR suppresses diet‐induced, TGF‐β driven hepatic fibrosis [41]. What was not apparent was whether prorenin—a soluble protein—would be displayed by EV in a way available to (p)RR. Prorenin is modified by high mannose carbohydrate polymers and is susceptible to cleavage by peptide: N‐glycosidase F (EndoF) [80]. Since this endopeptidase reduced the apparent molecular weight of all EV‐associated prorenin, vesicle‐associated prorenin is available to ligate platelet prorenin. Additionally, since deglycosylation suppressed aggregation, while renin enzymatic activity is insensitive to its glycosylation [81] and unglycosylated recombinant renin was an ineffective platelet agonist, this post‐translational modification may aid presentation of EV‐associated prorenin to platelet (p)RR.

Renin enzymatic activity and (p)RR signaling have the potential to contribute to hepatic fibrosis [82]. Renin mRNA expression is increased in the livers of cirrhotic patients [28, 29] and HSC express all components of the RAS system [83]. Accordingly, activated HSC generate and release the angiotensin II product of this system, and they respond to this cytokine through their G protein‐coupled AT1 receptor [28]. Thrombin, as well as the PDGF or EGF products of activated platelets, augments angiotensin II release that stimulates HSC growth, and thrombin‐induced angiotensin II formation by these HSC is sharply curtailed by renin inhibition. Hepatic (pRR) itself is profibrotic [84] and hepatocyte steatosis from mice fed high‐fat diets is attenuated by (p)RR inhibition [85]. Conversely, (p)RR knockdown attenuates steatosis and hepatic fibrosis [41, 86]. However, the way renin and prorenin production is controlled in any system remains largely undefined [27] and extra‐renal regulation of the two murine renin loci [87, 88, 89] differs in structure and function from the single human REN locus [90].

Ultimately, the effect of EV‐associated renin activity was to promote the formation of the extrinsic tenase and prothrombinase coagulation complexes on platelets that generate active thrombin. Our findings, then, establish a novel mode of entry into the stringently regulated external coagulation cascade. This intercellular interaction also provides a molecular understanding of the effects of therapeutic anti‐platelet, anti‐coagulation protease, and anti‐renin catalytic inhibitors in fibrotic liver disease [3, 16, 75, 76, 91]. Platelets express a limited proteome and a limited transcriptome that, along with their diminutive size, commonly excludes platelets from transcriptomic analysis. Yet, platelets are highly concentrated stores of a myriad of bioactive small molecule mediators, cytokines, and growth factors [92, 93] that include the TGF‐β that contributes to ConA‐induced hepatic fibrosis [47, 53]. We found deposition of TGF‐β in the livers of ConA‐exposed mice was not broadly distributed in the extracellular matrix, nor did it reside within hepatic or infiltrating inflammatory cells, but instead, its expression was focal and consistent with the degranulation of activated platelets. We conclude intrahepatic platelets are positioned to contribute to hepatic injury as a pre‐existing, non‐transcription dependent source of pro‐fibrotic TGF‐β. These observations provide a rational basis for the use of established anti‐thrombotic and anti‐platelet agonists in hepatic fibrosis and additionally provide a mechanistic basis to explore the anti‐fibrotic effects of direct renin inhibitors like Aliskiren.

Overall, this work identifies unanticipated actions of platelets residing in extravascular hepatic compartments to interact with activated HSC that initiate thrombin formation downstream of a novel route of entry into the tightly regulated coagulation cascade. This intercellular interaction is mediated by diffusible extracellular vesicles that functionally connect physically dispersed intrahepatic platelets with HSC in a low‐flow extravascular environment to support time‐dependent thrombin formation. This work also reveals how intrahepatic platelets can be activated in the early stages of hepatic fibrosis as an abundant source of TGF‐β able to contribute to fibrotic liver injury [11].

## Author Contributions

Rui Chen: conceptualization, original draft, experiments, data curation, formal analysis, methodology, software, validation, visualization, review and editing. Emily Huang: experiments, data curation, formal analysis, methodology, software, validation, visualization, and review. Xianfang Wu: methodology, experiments, data curation, formal analysis, and review. Megan McMullen: experiments, data curation, formal analysis, methodology, validation, and review. Xinjia Wang: experiments, data curation, formal analysis, validation, and review. Laura Nagy: conceptualization, methodology, formal analysis, and review. Scott Cameron: original draft, formal analysis, methodology, and review. Thomas McIntyre: conceptualization, original draft, formal analysis, methodology, validation, visualization, review and editing.

## Conflicts of Interest

The authors declare no conflicts of interest.

## Data Availability

All data are available in the main text of this article.
